# The mechanism of TiaoGanYiPi formula for treating chronic hepatitis B by network pharmacology and molecular docking verification

**DOI:** 10.1038/s41598-021-87812-9

**Published:** 2021-04-16

**Authors:** Xu Cao, Xiaobin Zao, Baiquan Xue, Hening Chen, Jiaxin Zhang, Shuo Li, Xiaobin Li, Shun Zhu, Rui Guo, Xiaoke Li, Yong’an Ye

**Affiliations:** 1 Dongzhimen Hospital, Beijing University of Chinese Medicine, Beijing, 100700 China; 2 Key Laboratory of Chinese Internal Medicine of Ministry of Education and Beijing, Dongzhimen Hospital, Beijing University of Chinese Medicine, Beijing, 100700 China; 3 Beijing University of Chinese Medicine, Beijing, 100102 China; 4 The First People’s Hospital of Jinzhou District, Dalian, 116100 China; 5 Institute of Liver Diseases, Beijing University of Chinese Medicine, Beijing, 100700 China

**Keywords:** Drug screening, Computational biology and bioinformatics, Immunology, Microbiology, Biomarkers, Gastroenterology, Hepatology, Hepatitis

## Abstract

The Chinese herbal formula TiaoGanYiPi (TGYP) showed effective against chronic hepatitis B (CHB) caused by hepatitis B virus (HBV) infection. Hence, we aimed to clarify the mechanisms and potential targets between TGYP and CHB. The active compounds and related putative targets of TGYP, and disease targets of CHB were obtained from the public databases. The key targets between TGYP and CHB were identified through the network construction and module analysis. The expression of the key targets was detected in Gene Expression Omnibus (GEO) dataset and normal hepatocyte cell line LO2. We first obtained 11 key targets which were predominantly enriched in the Cancer, Cell cycle and HBV-related pathways. And the expression of the key targets was related to HBV infection and liver inflammation verified in GSE83148 database. Furthermore, the results of real-time quantitative PCR and CCK-8 assay indicated that TGYP could regulate the expression of key targets including CCNA2, ABL1, CDK4, CDKN1A, IGFR and MAP2K1, and promote proliferation of LO2 cells. In coclusion, we identified the active compounds and key targets btween TGYP and CHB, and found that the TGYP might exhibite curative effect on CHB via promoting hepatocyte proliferation and inhibiting the liver inflammatory processes.

## Introduction

It has been a global public health problem for the hepatitis B virus (HBV) infection for so long^1^. The worldwide estimates suggested that more than 2 billion people have been infected with HBV, with 248 million of these people are chronic hepatitis B (CHB) patients. And about 15–25% of CHB patients died from cirrhosis or liver cancer^2^, which caused a substantial global burden^3^. In China, there are 20 ~ 30 million CHB patients, and the mortality rate of HBV-related complications is higher than that in the world^4^. The disease process of CHB is affected by both HBV and the host immune system, while the pathogenesis of CHB is complex and still unclear. Meanwhile, existing treatment options including nucleos(t)ide analogs (NAs) and interferons (IFN) are limited in their efficacy and scope of application. At present, the discontinuation of NAs lacked operability in clinc^5^, and oral antiviral drugs also had limitations in improving complications^6^. Meanwhile, the contraindications and side effects hindered the clinical application of IFN^7^.

For a long time, Chinese herbal medicine (CHM) plays a crucial role in CHB treatment and shows its superiority^8,9^. As for CHM prescriptions which contained a comlex compounds, often function with various multi-targeting and synergistic effects. Among CHM, the TiaoGanYiPi formula (TGYP) was used together with entecavir (ETV), and reported better efficacy for CHB treatment in our previous multi-center, double-blind, randomized placebo-controlled trial^10^. And in Chinese medicine theory, CHB is a complex-syndrome disease, and the TGYP is targeting at the core syndrome which is stagnation of Gan and deficiency of Pi qi, and damp-heat in the Gan and Dan^11^. However, the pharmacological mechanisms of TGYP regarding the therapeutic effect of CHB have not been fully explained. Here, we aimed to explore the relationship between CHB and TGYP, identify the key target genes and mechanisms of TGYP treatment on CHB through bioinformatics methods and in vitro experiments.

In this study, we first performed network pharmacology based on system biology and molecular docking, screened out 11 key target genes that exert essential functions during the treatment of TGYP on CHB. And the results of CCK-8 assay and real-time quantitative PCR (RT-qPCR) indicated that curative effect of TGYP could be related to promoting normal hepatocytes proliferation and regulation on the expression of partial key target genes. These results revealed the relationship between TGYP and CHB, further confirmed the effect of TGYP on hepatocytes, and indicated directions and targets for further research.

## Results

### Workflow of the study

**Figure 1:**
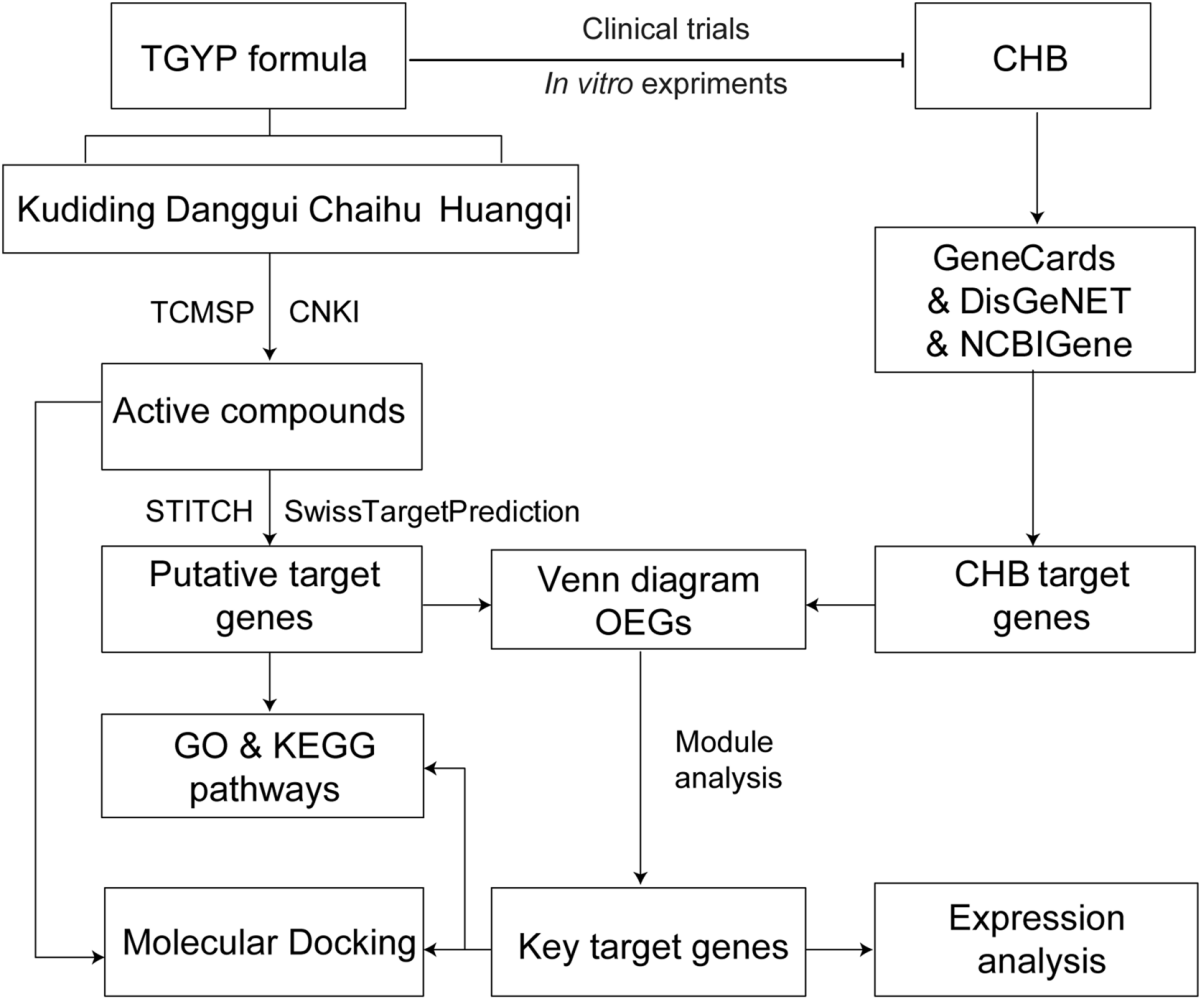
Flowchart of the study. TGYP target genes were firstly screened out according active compounds, and then performed with GO and KEGG pathways enrichment analysis. Next, CHB target genes were obtained from NCBI Gene, GeneCards and DisGeNET databases. The OGEs of TGYP and CHB target genes were isolated to identify key target genes. Then, the biologic process and expression of key target genes, interactions between of key compounds and key target genes were examined. At last, the expression of key genes were detected in GSE83148 database and in LO2 cells with TGYP treatment.

Based on the previous work, which showed the anti-HBV effect of TGYP, we designed the flowchart of this study (Fig. 1). As the flowchart showed, we first analyzed the four main herbs in TGYP by CNKI and TCMSP databases and then identified putative target genes by SwissTargetPrediction and STITCH analysis. Meanwhile, we analyzed and screened CHB disease targets through the GeneCards, DisGeNET and NCBI Gene databases. Next, we got the overlapped genes (OGEs) between the TGYP target genes and CHB desease targets to go further module analysis. Then, we obtained the key target genes for TGYP treatment on CHB and perfomed Gene Ontology (GO) and Kyoto Encyclopedia of Gene and Genome (KEGG) pathway analysis and molecular docking with active compound respectively. Finally, we detected the expression of key target genes in GSE83148 databse, and in LO2 cells with TGYP treatment by RT-qPCR, and CCK-8 assay was performed to detect proliferation of LO2 cells after TGYP treatment.

### Compound-putative targets network

To construct the compound-putative targets network of TGYP, we first analyzed the four main herbs in TGYP by CNKI and TCMSP databases and found 26 active components in Chaihu, 25 active compounds in Huangqi, 25 active compounds in Danggui, and 17 active compounds in Kudiding, respectively (Figure S1A). Among them, Chaihu and Huangqi have three commoncompounds, Chaihu and Danggui have two common compounds, the detailed information of all compounds was shown in Table S1. Next, according to the active compounds, we identified 775 candidate target genes by SwissTargetPrediction and 1,198 candidate target genes by STITCH analysis for TGYP (Table S2). After eliminating the redundancy, a total of 1,764 putative targets were collected (Figure S1B) and visualized as the compound-putative target network by Cytoscape (Figure S1C). The compound-putative target network showed 1,856 nodes and 4,941 edges, and according to Degree of the compound-putative target network, the top three ranked compounds were Isorhamnetin (Degree = 147), Quercetin (Degree = 145), and Kaempferol (Degree = 142), and thus we defined them as key compounds in TGYP.

### Functional enrichment of TGYP target genes

**Figure 2:**
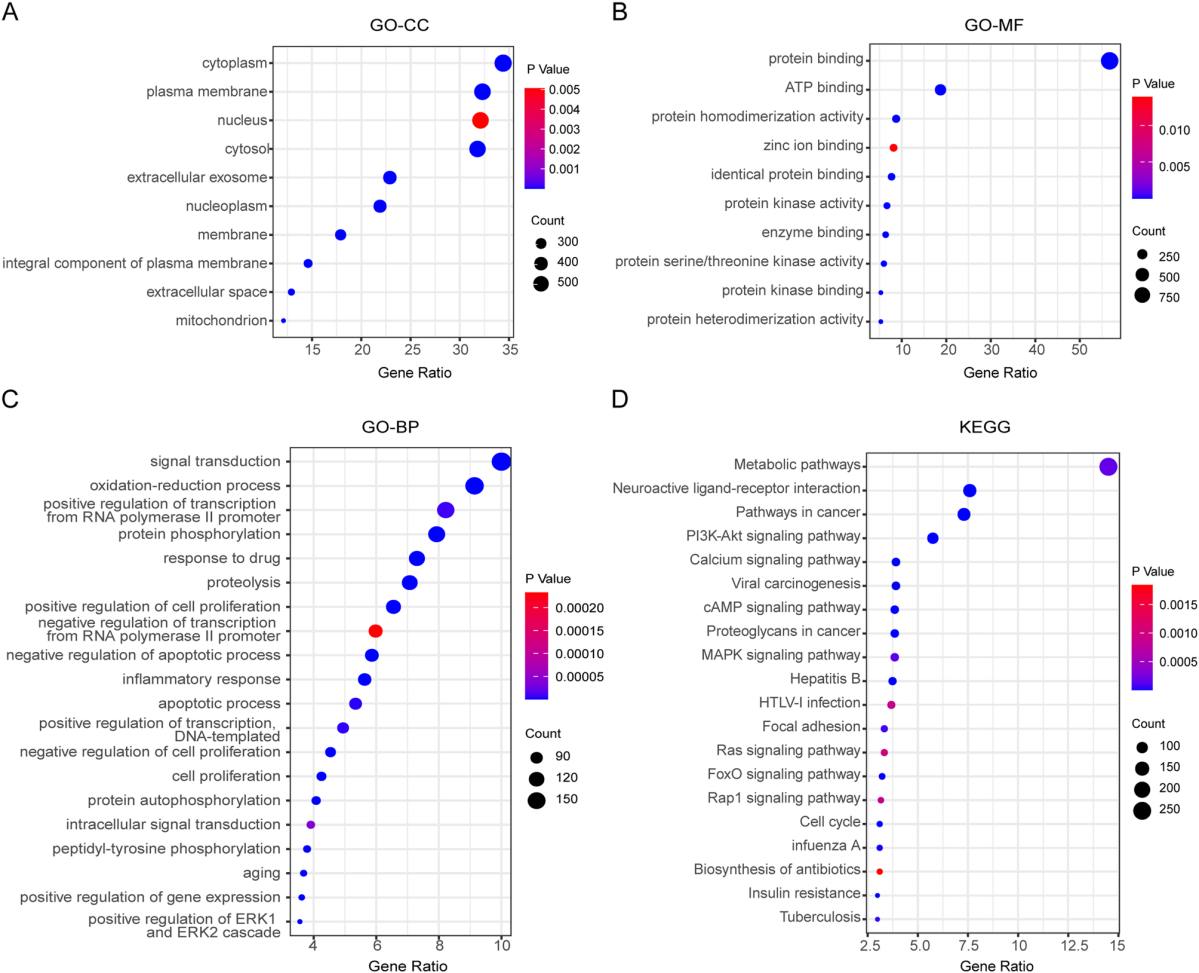
Functional enrichment of TGYP target genes. Functional annotation of active compounds related to putative targets. (**A**) Enriched cellular components. (**B**) Enriched molecular functions. (**C**) Enriched biological processes. (**D**) Enriched KEGG pathways. [Fig. 2 was drawn by Sangerbox tools (http://sangerbox.com/AllTools?tool_id=9697991)].

To explore the biological function of TGYP target genes, the GO and KEGG pathway enrichment analysis were performed by DAVID. The GO enrichment analysis revealed that the GO-Cellular Component (CC) of TGYP target genes were predominantly involved in the Cytoplasm, Plasma membrane and Nucleus (Fig. 2A); the GO- Molecular Function (MF) of TGYP target genes mainly included Protein binding, ATP binding, and Protein homo-dimerization activity (Fig. 2B); and the GO-Biological Process (BP) of TGYP target genes concentrated primarily on Signal transduction, Oxidation–reduction process, and Positive regulation of transcription from RNA polymerase II promoter (Fig. 2C). Meanwhile, the KEGG enrichment analysis suggested that TGYP target genes were primarily associated with Metabolic, Neuroactive ligand-receptor interactions and Cancer pathways (Fig. 2D). These results suggested that the TGYP formula had a wide range of regulation pathways and complex mechanisms.

### Identification and PPI network construction of OGEs

We analyzed and screened CHB disease targets, where we identified 8,037 targets in the GeneCards database, 415 targets in the DisGeNET database, and 236 targets in the NCBI Gene database, respectively (Table S3). And we subsequently obtained 8,083 disease targets of CHB after eliminating the redundancy and taking the union of these three sets (Figure S2A). Next, we examined the intersection of TGYP and CHB target genes and obtained 1,200 OGEs from the Venn diagrams tool (Figure S2B). After that, we constructed a protein–protein interaction (PPI) network of OGEs and analyzed their corresponding secondary proteins from the STRING tool (Figure S2C). The PPI network results showed the interactions between OGEs, and there were highly tight connections existed.

### Identification of the key target genes through modules analysis

**Figure 3:**
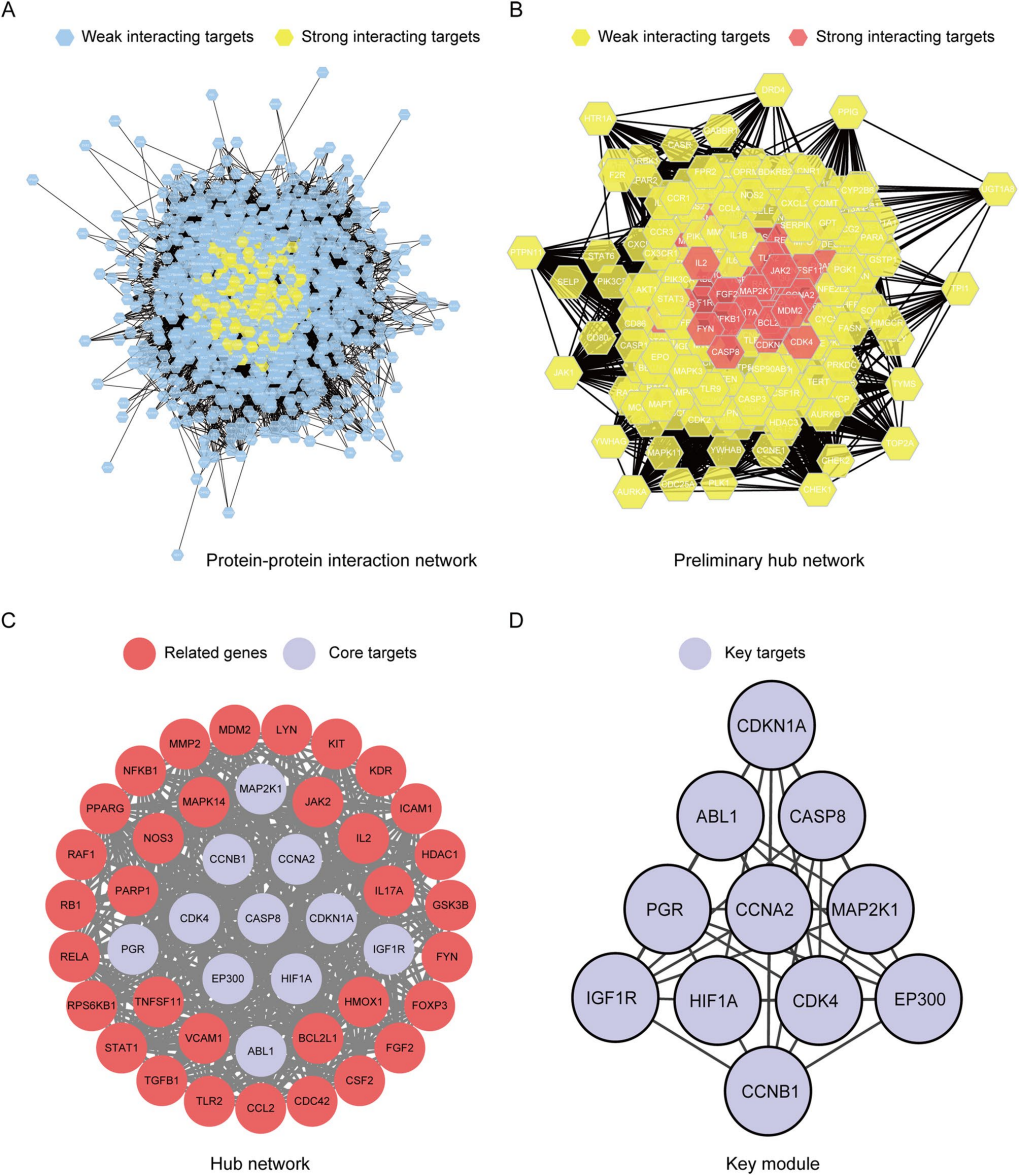
Identification of the key target genes through modules analysis. (**A**) Protein-protein interaction network. Blue hexagons represent weak interacting target genes, yellow hexagons represent strong interacting target genes; (**B**) Preliminary hub network. Yellow hexagons represent weak interacting target genes, red hexagons represent strong interacting target genes; (**C**) Hub network. Red circles represent related target genes, purple circles represent core target genes; (**D**) Key module. Purple circles represent key target genes. [Fig. 3 was drawn by Cytoscape 3.8.0 (https://www.cytoscape.org/)].

Since there was one gene lacked a corresponding protein structure and seven proteins did not interact with others based on PPI network, the remaining 1,192 proteins from STRING data were imported into the Cytoscape to further obtain interaction networks (Fig. 3A). Filtering with the network topological parameters, when the Degree is greater than twice the median (Degree > 78), we get the preliminary hub network (Fig. 3B). Based on the fact that Betweenness Connectivity (BC), Closeness Connectivity (CC), Degree Connectivity (DC), Local Average Connectivity (LAC), Neighbor Connectivity (NC), and Subgraph Centrality (SC); are above the median (BC > 0.001, CC > 0.574, DC > 61, LAC > 38.452, NC > 89.011, and SC > 5.964E + 35), we identified 44 highly connected nodes as hub networks (Fig. 3C). The MCODE plugin in Cytoscape was used for a module analysis of the targets in the hub network. The module analysis identified four clustering modules, and we chose the key module, which had the highest network heterogeneity and network centralization at 0.242 and 0.333, respectively. The clustering key module we obtained represents a key targets for treating CHB with TGYP. We acquired a dense region network of 11 key targets (Fig. 3D), including ABL Proto-Oncogene 1, Non-Receptor Tyrosine Kinase (ABL1), Caspase 8 (CASP8), Cyclin A2 (CCNA2), Cyclin B1 (CCNB1), Cyclin Dependent Kinase 4 (CDK4), Cyclin Dependent Kinase Inhibitor 1A (CDKN1A), E1A Binding Protein P300 (EP300), Hypoxia Inducible Factor 1 Subunit Alpha (HIF1A), Insulin Like Growth Factor 1 Receptor (IGF1R), Mitogen-Activated Protein Kinase Kinase 1 (MAP2K1), and Progesterone Receptor (PGR).

### Functional enrichment analysis of 11 key target genes

**Figure 4:**
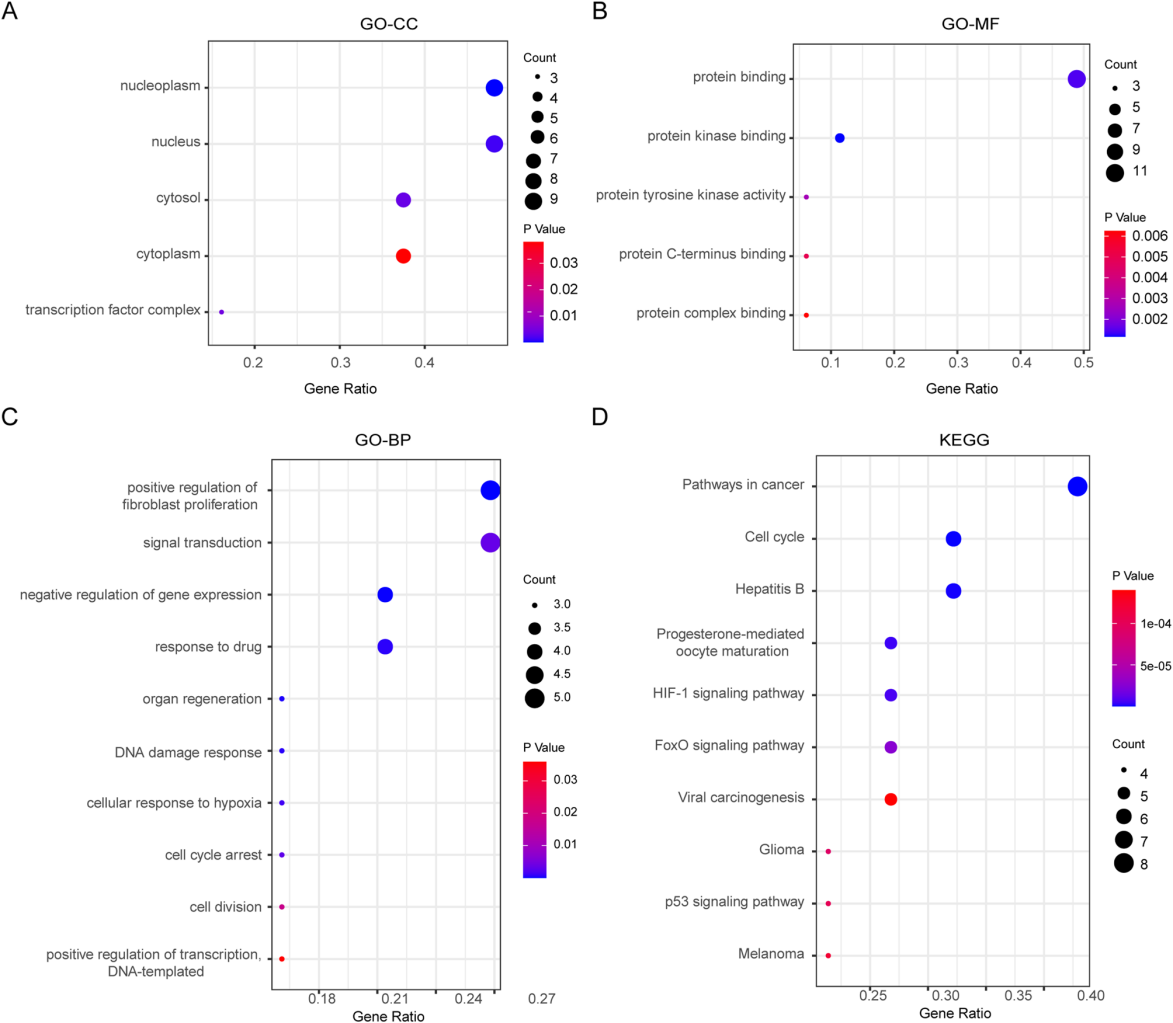
Functional enrichment of the key target genes. (**A**) Enriched cellular components. (**B**) Enriched molecular functions. (**C**) Enriched biological processes. (**D**) Enriched KEGG pathways. [Fig. 4 was drawn by Sangerbox tools (http://sangerbox.com/AllTools?tool_id=9697991)].

Functional enrichment analysis of the key target genes was performed after data screening to further understand the biological behaviors (Table S4). The GO-CC analysis showed that the key targets were mainly enriched in the Nucleoplasm, Nucleus, and Cytosol (Fig. 4A). GO-MF analysis suggested that the key targets were strongly associated with Protein binding, Protein kinase binding, and Protein tyrosine kinase activity (Fig. 4B). And GO-BP analysis showed that the key targets were significantly related to Positive regulation of fibroblast proliferation, Signal transduction, and Negative regulation of gene expression (Fig. 4C). The pathway enrichment results suggested that the key targets were mostly involved in pathways of Cancer, Cell cycle, Hepatitis B (Fig. 4D). In general, these biological processes and signaling pathways might be linked to beneficial effects of TGYP against CHB. And TGYP might have an effect on CHB-related complications, such as liver fibrosis and liver cancer, which require further exploration.

### The molecular docking of key compounds and key target genes

**Figure 5:**
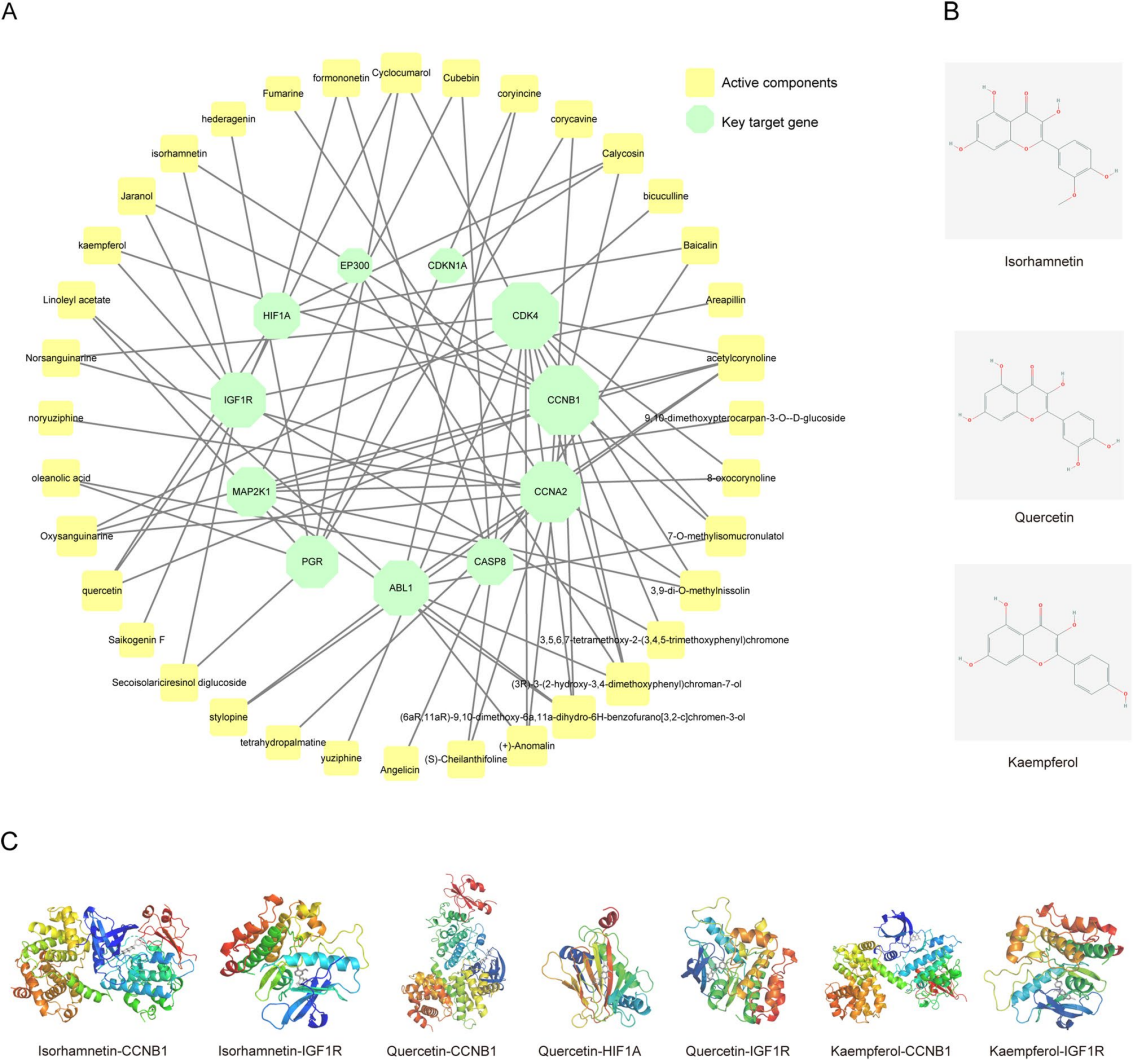
The molecular docking of key compounds and key target genes. (**A**) Interaction network of key target genes and corresponding active compounds of prediction. The green octagons represent key target genes, the yellow squares represent active compounds of prediction. The size of the icon was set according to the degree of the network. [drawn by Cytoscape 3.8.0 (https://www.cytoscape.org/)]. (**B**) The structure of key compounds. (**C**) The molecular docking of key compounds and key target genes.

To better understand the interactions between the compounds and target genes, we constructed an interaction network between the key target genes and corresponding active compounds in TGYP (Fig. 5A). According to the compound-putative analysis results, the key compounds were Isorhamnetin, Quercetin, and Kaempferol. Their 2D structures were showed in Fig. 5B. To further explore the interactions between key compounds and key target genes, we performed molecular docking by AutoDock Vina software (Fig. 5C). A grid box size of 30 × 30 × 30 points with a spacing of 1.0 Å between grid points was generated to cover almost the entire favorable protein-binding site. And the X, Y and Z centers were adjusted according to different macromolecular forms, mainly with the molecular active pocket as the center, and a few center on macromolecule. The results of docking capabilities of the protein-compounds included key target genes docked with key compounds and the most probable active compounds, respectively. Besides, ETV and corresponding targets of TGYP were docked to get affinity baseline data and the results showed that only 2 baseline groups yielded higher values than the TGYP group. The pair with the lowest binding affinity (-6.6 kcal/mol) was Calycosin and CDKN1A, whereas the pairs with the second- and third-lowest binding affinity (-6.8 and -7.1 kcal/mol) were Saikogenin F and HIF1A and Cubebin and CASP8 docking, respectively. The results of molecular docking are shown in Table 1, and the above results further indicated the regulation mechanism of TGYP on the key targets on the protein level.

**Table 1:** The docking scores of key compounds and key target genes.

Targets	PBD ID	Compounds	Affinity (kcal/mol)	Baseline affinity (ETV, kcal/mol)
ABL1	6BL8	( +)-Anomalin	−8.4	−6.9
CASP8	6PX9	Baicalin	−8.1	−5.7
CASP8	6PX9	Cubebin	−7.1	−5.7
CCNA2	1E9H	corycavine	−10.1	−7.6
CCNB1	6GU2	Calycosin	−7.9	−6.7
CCNB1	6GU2	quercetin	−7.5	−6.7
CCNB1	6GU2	Isorhamnetin	−7.6	−6.7
CCNB1	6GU2	Kaempferol	−8.0	−6.7
CDK4	5FWP	( +)-Anomalin	−8.3	−9.4
CDKN1A	6P8H	Calycosin	−6.6	−5.5
EP300	6PF1	(3R)-3-(2-hydroxy-3,4-dimethoxyphenyl)chroman-7-ol	−8.2	−6.9
HIF1A	3HQU	quercetin	−7.7	−7.1
HIF1A	3HQU	Saikogenin F	−6.8	−7.1
IGF1R	3D94	quercetin	−8.3	−6.9
IGF1R	3D94	kaempferol	−8.2	−6.9
IGF1R	3D94	Isorhamnetin	−8.1	−6.9
MAP2K1	3EQF	8-oxocorynoline	−12.7	−7.5
PGR	1A28	Secoisolariciresinol diglucoside	−8.1	−6.7
PGR	1A28	hederagenin	−7.4	−6.7

### Expression of the key target genes in CHB liver tissues and correlation with liver inflammation

**Figure 6:**
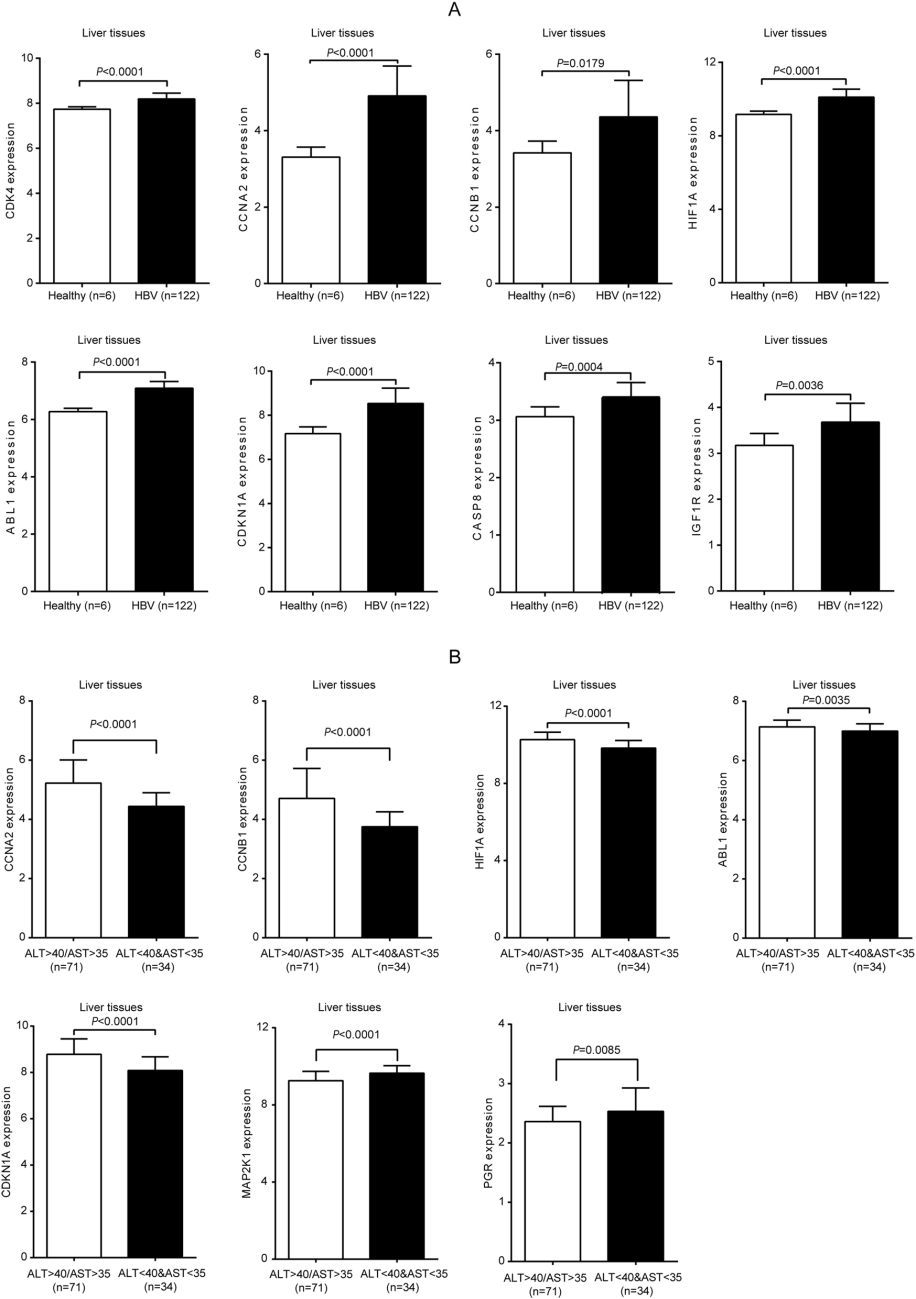
Expression of key genes in CHB liver tissues and correlation with liver inflammation. (**A**) The upregulation of key target genes in CHB liver tissues. (**B**) The key target genes expression was correlated to liver inflammation in CHB. For the comparison of quantitative data between groups, the Student’s t-test is used. Differences in means were considered statistically significant at P < 0.05.

Furthermore, CHB patients with significant liver inflammation could benefit from improved antiviral effect^12^. Thus, we further analyzed the key target genes expressions in CHB liver tissues and their correlations with liver inflammation in the GEO dataset GSE83148. Compared to healthy controls, some key target genes were significantly higher expressed in CHB patients (P < 0.05), including ABL1, CASP8, CCNA2, CCNB1, CDK4, CDKN1A, HIF1A, and IGF1R (Fig. 6A). We further compared the expression of the key target genes between 71 patients with abnormal serum alanine aminotransferase (ALT) or aspartate aminotransferase (AST) levels (ALT ≥ 40 or AST ≥ 35) and 34 patients with normal serum ALT and AST levels (ALT < 40 and AST < 35). And the results showed that ABL1, CCNA2, CCNB1, CDKN1A, and HIF1A were significantly higher expressed in the abnormal group than normal group, whereas MAP2K1 and PGR expressions were reversed (Fig. 6B). These results indicate that expression of these key genes might be related to CHB process and TGYP might release the disease status and the inflammatory state by regulating the expression of these genes.

### TGYP regulates the key target genes expression and increases proliferation of hepatocyte

**Figure 7:**
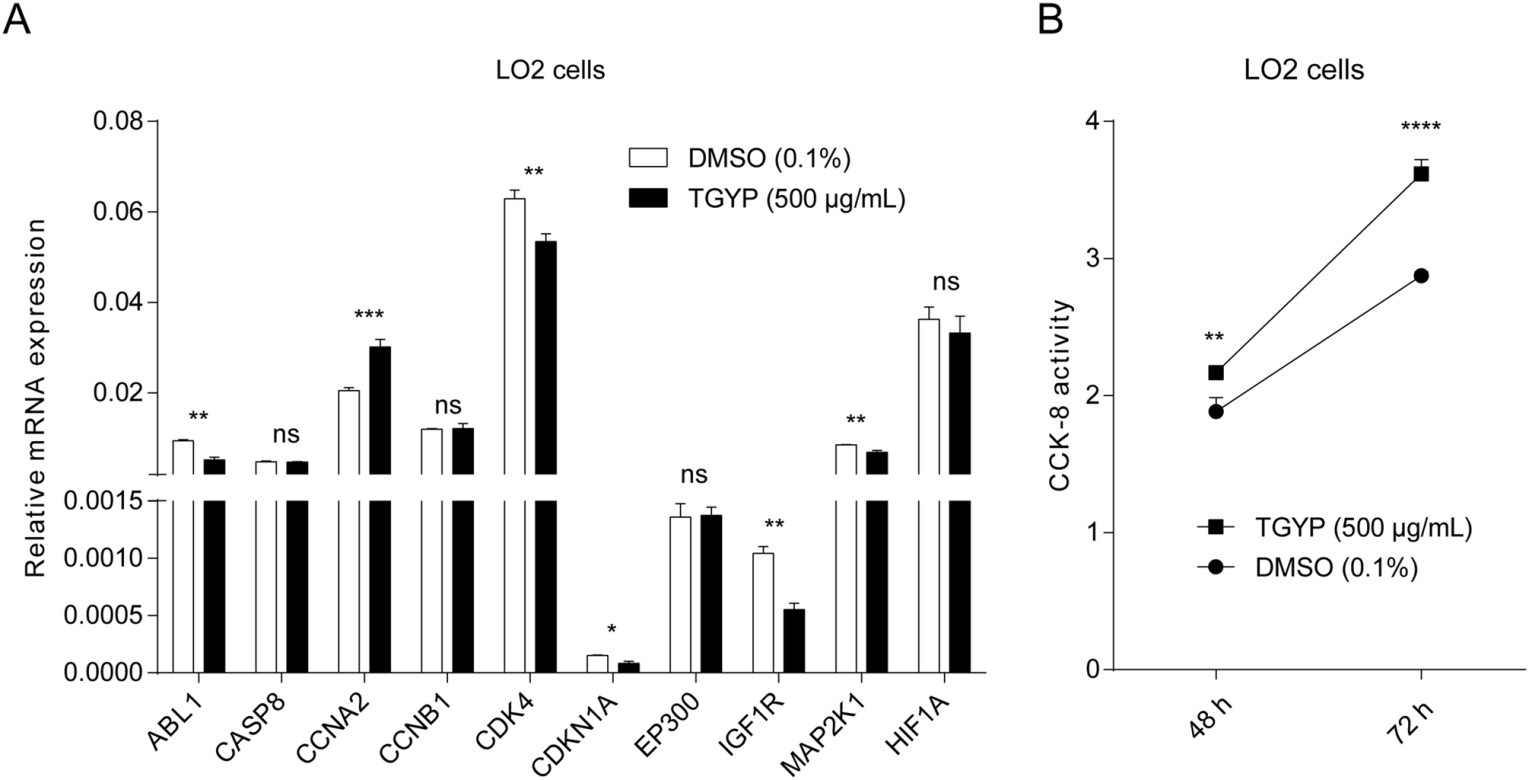
TGYP regulates the key target genes expression and promotes proliferation of LO2 cells. (**A**) LO2 cells were treated with TGYP for 48 h and the mRNA expression of key genes were detected by RT-qPCR. (**B**) LO2 cells were treated with TGYP, and the cell vibility was detected after 48 h and 72 h. For the comparison of quantitative data between groups, the Student’s t-test is used. Differences in means were considered statistically significant at P < 0.05. Significance levels are: *, P < 0.05; **, P < 0.01; ***, P < 0.001; ****, P < 0.0001; ns, not significant.

To further confirm the regulatory effect of TGYP on the key target genes, we treated LO2 cells with TGYP and detected the expression of key target genes by RT-qPCR. The results showed that among the key target genes, CCNA2 expression was upregulated, and ABL1, CDK4, CDKN1A, IGFR and MAP2K1 expression was downregulated after TGYP treatment, while the expression of PGR was below the limit of detection (Fig. 7A). Cause these regulated genes were closely related to cell proliferation, to confirm the effect of TGYP on the hepatocyte avibility, we treated LO2 cells with TGYP and the results showed that the CCK-8 activity was significantly increased after TGYP treatment compared to control (Fig. 7B). These results indicated that the expression of CCNA2, ABL1, CDK4, CDKN1A, IGFR and MAP2K1 could be regulated and play an important role during TGYP treatment on CHB.

## Discussion

CHB is a refractory disease which is closely related to the occurrence and development of liver fibrosis and liver cancer, and causes great threats to public health^13^. At present, NAs and IFNs are the first options for CHB treatment, whereas both of them have shortcomings. NAs cannot eliminate HBV cccDNA in hepatocytes to achieve complete cure, while IFNs has many contraindications and side effects^14^. In contrast, traditional Chinese medicines have unique advantages against CHB, such as better safety profile and lower price^8^. Our preliminary study confirmed the capability and safety of TGYP for improving HBeAg loss after combination with ETV, thereby reducing disease-related risks in long terms^10^. For TGYP, the main components are Chaihu, Diding, Huangqi and Danggui and it is noteworthy that other studies have reported that Huangqi could relieve HBV induced hepatitis by inhibiting NF-κB signaling^15^, and Chaihu could promote HBeAg clearance by inhibiting HBV replication^16,17^. Combined these studies, it suggested that multiple active compounds of TGYP could have inhibitory effect on HBV in different pathways. It is necessary to explore internal mechanisms to find more treatment targets.

To further determine the mechanism of TGYP on CHB, we performed network pharmacology and database mining in this study. We firstly screened out TGYP target genes based on drug active compounds of the four main herbs. Among the active molecules, the top three active compounds with the highest network connectivity were Isorhamnetin, Quercetin, and Kaempferol. These compounds were observed with inhibiting liver inflammation or anti-HBV effects, such as Isorhamnetin could significantly reduce serum levels of liver enzymes and pro-inflammatory cytokines in rats^18^; Quercetin could strongly inhibit HBeAg synthesis and slightly anti-HBV surface antigen activity^19^, and Kaempferol could significantly reduce TNF-α expression and inflammatory responses^20^. These results suggested that TGYP may exert anti-HBV function via these active compounds and their target genes and pathways. We then combined CHB related genes to construct the interaction network of TGYP and CHB, where 11 key target genes were identified. Molecular docking analysis predicted direct binding between core active compounds and key target genes. We also found that in the GSE83148 database, compared with healthy controls, the expression of key target genes including ABL1, CASP8, CCNA2, CCNB1, CDK4, CDKN1A, HIF1A, and IGF1R were all upregulated in liver tissues of CHB patients. Thus, the core active compounds of TGYP might exert anti-CHB functions by regulating the expression and protein activities of the key target genes.

Additionally, we found that the key target genes were mainly enriched in Cancer, Cell cycle, and Hepatitis B signaling pathways using the KEGG pathway enrichment analysis. Specifically, among the 11 key target genes, CCNB1, CDKN1A, EP300, ABL1, CDK4, and CCNA2 are enriched in the Cell cycle pathway, and previous studies have shown that the expression of these factors could regulate the cell cycle in varying degrees, promote or inhibit cell proliferation^21–26^. Hepatocyte proliferation has been reported to accelerate during HBV infection^27^, leading to the downregulation of HBV receptor NTCP in hepatocytes, which contributes to HBV cccDNA loss and further accelerates HBV clearance^12,28^. Our study suggested that TGYP might exert anti-HBV effects by regulating the cell-cycle-related genes and promoting infected hepatocyte proliferation. However, the specific regulatory mechanism needs to be further investigated in HBV replication models.

The key target genes enriched in the Hepatitis B pathway include MAP2K1 and CASP8. For MAP2K1, studies have shown that activation of the Ras-Raf-MAP2K1-ERK pathway could inhibit HBV replication^29,30^, while HBV infection could induce IL-23 and IL-6 expression in hepatocytes through MAP2K1, which could inhibit anti-HBV immune response^31,32^. In addition, HBx could induce hepatocyte apoptosis by activating the MAPK pathway, which was also an important cause of CHB^33^. For CASP8, its expression on peripheral blood mononuclear cells was positively correlated with HBV DNA level in CHB patients^34^. The combination of Caspase-8 and TNFAIP8L2 could inhibit the activities of AP-1 and NF-κB, which in turn promoted Fas-induced apoptosis of immune cells^35^. Moreover, HBx could increase the expression level of caspase-8 and promote apoptosis induced by exogenous pathways mediated by death receptors^36^. Meanwhile, studies have shown that HBV infection could inhibit the innate immune pathways in hepatocytes and the liver's adaptive immune system, which was critically important for the development of CHB^37^. In the current study, we found the expression of MAP2K1 was correlated with the severety of liver inflammation and TGYP treatment could downregulate its expression. It is hypothesized that the TGYP might release the progression of CHB by regulating the immune response in the liver, which needs to be further investigated in animal models.

Finally, IGF1R and HIF1A genes enriched in Cancer pathways were closely related to the occurrence and development of HBV-related HCC. Studies have shown that HBx could trans-activate endogenous IGFIR expression and promote cell mitosis, thereby promoting the proliferation of liver cancer cells^38^. Meanwhile, HBx could enhance the transcriptional activity of HIF1A, thus promoting para-tumor angiogenesis^39,40^. Therefore, abnormal upregulation of IGF1R and HIF1A could promote the occurrence and metastasis of HBV-associated liver cancer. Recent studies have shown that although NAs may inhibit HBV replication in hepatocytes, they cannot completely reverse HBV-induced signal transduction pathway activation or cell cycle regulatory protein change^41^. Those infected hepatocytes were still in transition to cancer cells. In contrast, our preliminary study demonstrated the superiority of integrative therapy regarding HBeAg clearance, and in this study, we found that TGYP could downregulate the expression of IGF1R, thereby playing a role in the inhibition of HBV-related HCC. The above results suggested TGYP may exert beneficial to prevent the risk of HCC in CHB patients. These results need to be validated in a larger sample, whilst the mechanisms need to be explored with in vitro and in vivo models.

To sum up, a network between active compounds of TGYP and CHB related genes was constructed; and further network pharmacology, molecular docking verification and in vitro expriments demonstrated that this CHM formula might exert an anti-CHB effect through upregulating hepatocyte proliferation, releasing liver inflammation, and enhancing immune response. These findings could provide further insights into the mechanisms of CHM-based therapy on CHB and assist in screening potential therapeutic targets in the future.

## Methods

### Collection of chemical compounds in TGYP

Compounds of the main herbs in TGYP, including Chaihu, Huangqi, Danggui, and Kudiding were searched in CNKI and TCMSP database (http://tcmspw.com/tcmsp.php). The active compounds in TCMSP were mainly filtered by integrating the pharmacokinetic properties comprising oral bioavailability (OB) ≥ 30%^42^, drug-likeness (DL) ≥ 0.18^43^, as well as half-life (HL) ≥ 4 h. The corresponding compounds chemical 2D structure SDF and canonical SMILES acquired from PubChem database (https://pubchem.ncbi.nlm.nih.gov). Data formats were transformed with Open Babel GUI software^44^. The Venn diagram was charted by the Venn tool (http://bioinformatics.psb.ugent.be/webtools/Venn/).

### Prediction of putative targets in TGYP

The canonical SMILES or SDF of the active compounds were imported into the SwissTargetPrediction (http://www.swisstargetprediction.ch/) and the STITCH databases (http://stitch.embl.de/), respectively. Putative targets of TGYP were collected under the following conditions: selecting the species is homo sapiens. The probability value of each potential target listed in the SwissTargetPrediction database was used to investigate the accuracy of the current predictions, whose probability value ≥ 0.1 was identified. In addition, the potential target proteins with confidence score ≥ 0.15 in the STITCH database.

### Identification of CHB targets

The target genes related to CHB were gathered from followed three databases. The GeneCards database (https://www.genecards.org/), the DisGeNET database (https://www.disgenet.org/) and the NCBI gene database (https://www.ncbi.nlm.nih.gov/gene/). The search condition was using the keyword “Chronic Hepatitis B” and selecting the organisms “Homo sapiens” from the three platforms. The above gene ID was identified and standardized in the UniProt database (https://www.uniprot.org). We used Retrieve/ID mapping tool of UniProt to convert identifiers, which are of a different type to UniProt identifiers.

### Network construction

The overlapped genes (OGEs) of the compound-putative targets and disease targets were charted by the Venn tool. We inputted the OGEs into the STRING database (https://string-db.org/) with confidence scores ≥ 0.4 and the species limited to “Homo sapiens”, and exported protein–protein interaction (PPI) data. Analyzed the PPI data and constructed network by the Cytoscape 3.8.0 (https://cytoscape.org/), and the non-connection genes were removed. We use the Analyzer plugin to analyze the PPI network and get the degree, through taking the over a double median of degree to get the preliminary hub network^45^. Then, using the CytoNCA plugin to analyze the preliminary hub network to get network topological parameters: BC, CC, DC, LAC, NC and SC. We took the excess median of BC, CC, DC, LAC, NC, and SC to obtain hub network^46^. The MCODE application calculated the hub network to get the dense regions^47^. The genes in dense regions from MCODE is called key targets.

### Analysis of GO and KEGG pathway

The screened out genes were analyzed by the GO function analysis included BP, MF and CC^48^, and the KEGG analysis (https://www.genome.jp/kegg/). The GO function enrichment and KEGG pathway enrichment were analyzed by DAVID database (https://david.ncifcrf.gov/). The filtering of retrieval results is with a threshold value of p < 0.05 and count in descending order. The value of P reflected the significance of protein biological function, and the purpose of filtering is to select the most enriched biological annotation from a given list of genes. Bubble Diagrams were drawn by Sangerbox tools (http://sangerbox.com/AllTools?tool_id=9697991).

### Molecular docking simulation

Molecular docking simulation was performed to verify the critical components' binding ability to the key targets and explore their accurate binding modes. The macromolecular protein target receptors were acquired from the RCSB PDB database (https://www.rcsb.org). Moreover, the 2D structures of the compounds were obtained from the PubChem Database. AutoDock Vina could markedly improve the average accuracy of the combined mode prediction^49^. Thereby the Molecular docking simulation was performed by AutoDock Vina software, and the result was visualized by PyMOL software^50^. In this analysis, the value of the vina score indicates the binding activity between a compound and a protein, and the lower the vina score, the more stably the compound binds to the protein.

### Verification the expression of key genes

The expression of key genes was verified in the GEO database (http://www.ncbi.nlm.gov/geo/). The keywords were set as "hepatitis B" and "homo sapiens", the tissue type was limited to liver tissue, and the gene expression data of GSE83148^51^ was downloaded from the GEO database. The platform of dataset GSE83148 is the GPL570 (HG-U133_Plus_2) Affymetrix Human Genome U133 Plus 2.0 Array, which includes 122 patients with CHB and 6 healthy liver samples, and includes 71 patients with abnormal transaminase and 34 patients with normal transaminase, which threshold of ALT was 40 and the threshold of AST was 35.

### Cell line and Chinese herbal compounds

LO2 cells are regarded as human normal liver cell and maintained in dulbecco's modified eagle medium (DMEM) supplemented with 10% fetal bovine serum (FBS), 100 U/mL penicillin, 100 µg/mL streptomycin and 5% CO2. For the acquisition of Chinese herbal compounds, the granules of TGYP formula were obtained and authenticated by the Nanning Peili Pharmaceutical Co., Ltd. The granules was dissolved in DMSO to 500 mg/mL and stored at -20 °C. The TGYP solution was added into cell culture with 500 µg/mL and use DMSO as control.

### Real-time quantitative PCR (RT-qPCR)

The 5 × 10^5^ LO2 cells were seeded in 6-well plates. After incubated for 24 h, the cells were treated with TGYP (500 μg/ml) for 48 h. Total RNAs were extracted using the RaPure Total RNA Mini Kit (Magen, CN) according the manufacturer’s instructions. The reverse transcription of total RNA to cDNA was performed with qPCR RT Master Mix kit (TOYOBO, JAN). RT-qPCR was performed using the Real-time PCR Detection System (Agilent Technologies, US) with the SYBR Green Real-time PCR Master Mix (TOYOBO, JAN). The primers used in this study are provided in Supplementary Table S5, using GAPDH as internal control gene. The experiments were performed in triplicate and repeated 3 times.

### Cell viability assay

The 5 × 10^3^ LO2 cells were seeded in 96-well plates with four duplications, and after TGYP treatment, CCK-8 assay kit (Solarbio, CN) was carried out to assess the ability of cell growth through measuring the absorbance at the wavelength of 450 nm by the TECAN infinite M200 Multimode microplate reader (Tecan, Mechelen, Belgium).

### Statistical analysis

Data for graphing was processed with GraphPad Prism 8.0 software. Statistical analyses were performed using the SPSS 25.0 statistical software package. For the comparison of quantitative data between groups, the Student’s t-test is used. Differences in means were considered statistically significant at P < 0.05. Significance levels are: *, P < 0.05; **, P < 0.01; ***, P < 0.001; ****, P < 0.0001; ns, not significant.

## Supplementary Information

Supplementary Information 1.

Supplementary Information 2.

Supplementary Information 3.

Supplementary Information 4.

Supplementary Information 5.

Supplementary Information 6.

## Data Availability

All the relevant data is provided within the paper and its supporting information files. The datasets analyzed during the current study available from the corresponding author on reasonable request.

## Abbreviations

HBV: Hepatitis B virus
CHB: Chronic hepatitis B
NAs: Nucleos(t)ide analogs
IFN: Interferons
CHM: Chinese herbal medicine
TGYP: TiaoGanYiPi
RT-qPCR: Real-time quantitative PCR
OGEs: Overlapped genes
GO: Gene ontology
KEGG: Kyoto encyclopedia of gene and genome
CC: Cellular component
MF: Molecular function
BP: Biological process
PPI: Protein–protein interaction
BC: Betweenness connectivity
CC: Closeness connectivity
DC: Degree connectivity
LAC: Local average connectivity
NC: Neighbor connectivity
SC: Subgraph centrality
ABL1: ABL proto-oncogene 1, non-receptor tyrosine kinase
CASP8: Caspase 8
CCNA2: Cyclin A2
CCNB1: Cyclin B1
CDK4: Cyclin dependent kinase 4
CDKN1A: Cyclin dependent kinase inhibitor 1A
EP300: E1A binding protein P300
HIF1A: Hypoxia inducible factor 1 subunit alpha
IGF1R: Insulin like growth factor 1 receptor
MAP2K1: Mitogen-activated protein kinase kinase 1
PGR: Progesterone receptor
ALT: Alanine aminotransferase
AST: Aspartate aminotransferase
ETV: Entecavir
OB: Oral bioavailability
DL: Drug-likeness
HL: Half-life
DMEM: Dulbecco's modified eagle medium
FBS: Fetal bovine serum
ns: Not significant
