# Supplementary material for: The mechanism of TiaoGanYiPi formula for treating chronic hepatitis B by network pharmacology and molecular docking verification
Source: Sci Rep. 2021 Apr 16;11:8402. doi: 10.1038/s41598-021-87812-9 (PMC8052433; doi:10.1038/s41598-021-87812-9)

**The mechanism of TiaoGanYiPi formula for treating chronic hepatitis B by network pharmacology and molecular docking verification**

Xu Cao^1, 3*^, Xiaobin Zao^1, 2*^, Baiquan Xue^4*^, Hening Chen^1, 3^, Jiaxin Zhang^1, 3^,

Shuo Li^1, 3^, Xiaobin Li^1, 3^, Shun Zhu^1, 3^, Rui Guo^3^, Xiaoke Li^1, 5#^ & Yong’an Ye^1, 5#^

^1^ Dongzhimen Hospital, Beijing University of Chinese Medicine, Beijing 100700, China;

^2^ Key Laboratory of Chinese Internal Medicine of Ministry of Education and Beijing, Dongzhimen Hospital, Beijing University of Chinese Medicine, 100700 Beijing, China;

^3^ Beijing University of Chinese Medicine, Beijing 100102, China;

^4^ The First People's Hospital of Jinzhou District

^5^ Institute of Liver Diseases, Beijing University of Chinese Medicine, Beijing 100700, China;

* These authors contributed equally: Xu Cao, Xiaobin Zao and Baiquan Xue

^#^Correspondence should be addressed to Yong’an Ye and Xiaoke Li

**Figure S1.** **Compound-Putative Targets Network.** (A) The Venn diagram for active compounds of four herbs in TGYP. Blue oval represents Chaihu, teal oval represents Kudiding, green oval represents Danggui, red oval represents Huangqi; (B) The Venn diagram for compound-putative target genes. Red circle represents STITCH, Blue circle represents SwissTargetPrediction [(A) and (B) drawn by Venn tool (<http://bioinformatics.psb.ugent.be/webtools/Venn/>)]; (C) The visualized compound-putative target network of TGYP. White diamonds represent herb in TGYP, teal rectangle represent compound target genes, green circles represent the components in Chaihu, red circles represent the components in Huangqi, blue circles represent the components in Kudiding, yellow circles represent the components in Danggui, A1 represents the components Quercetin, A2 represents the components Isorhamnetin, A3 represents the components Kaempferol, B1 circle represents the components stigmasterol, B2 circle represents the components psoralen [drawn by Cytoscape 3.8.0 (<https://www.cytoscape.org/)>].


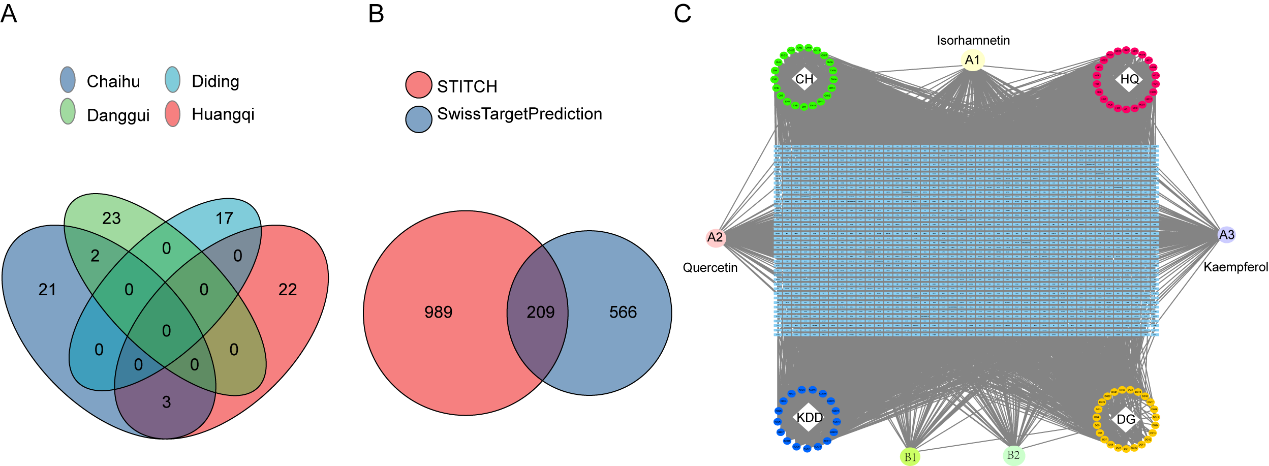


**Figure S2. OGEs: Intersecting Compound-Putative Targets and Disease targets in CHB.** (A) The Venn diagram for CHB related targets. Blue circle represents GeneCards, red circle represents DisGeNET, green circle represents NCBI Gene; (B) Venn diagram for OGEs of TGYP and CHB targets. Red circle represents compound target genes, blue circle represents disease target genes, purple circle represents OGEs [(A) and (B) drawn by Venn tool (<http://bioinformatics.psb.ugent.be/webtools/Venn/>)]; (C) PPI network of OGEs [drawn by STRING database ([https://string-db.org/)](https://www.cytoscape.org/))].


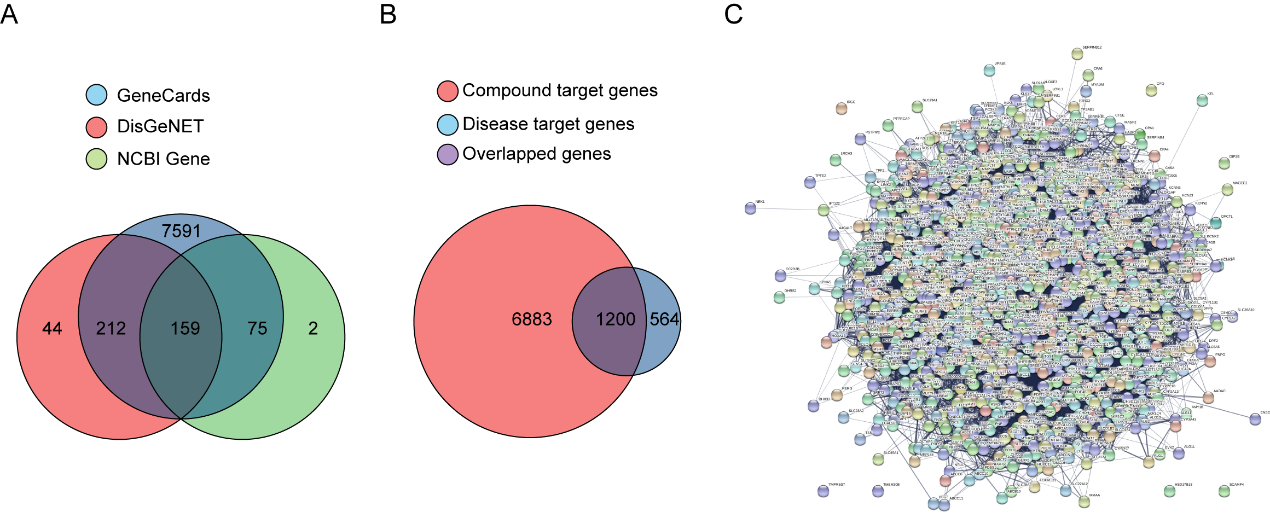

Supplement: Supplementary file 1 — Supplementary Information 1. [file 41598_2021_87812_MOESM1_ESM.docx]
